# The comparison of automated clustering algorithms for resampling representative conformer ensembles with RMSD matrix

**DOI:** 10.1186/s13321-017-0208-0

**Published:** 2017-03-23

**Authors:** Hyoungrae Kim, Cheongyun Jang, Dharmendra K. Yadav, Mi-hyun Kim

**Affiliations:** 1 Department of Pharmacy, College of Pharmacy, Yeonsu-gu, Incheon, Republic of Korea; 2 Gachon Institute of Pharmaceutical Science, Gachon University, Yeonsu-gu, Incheon, Republic of Korea; 3 Department of Data Management, KEIS, 56 Mullae-ro 20-gil, Yeongdeungpo-gu, Seoul, Republic of Korea

**Keywords:** Conformer ensemble, 3D shape-based alignment, k-Means clustering of multidimensional scaled RMSD values (RCKmeans), Hierarchical clustering with dynamic tree cut (RCDT), Linear kernel PCA (RCPCA), Nonlinear kernel PCA (RCPCA_RBF)

## Abstract

**Background:**

The accuracy of any 3D-QSAR, Pharmacophore and 3D-similarity based chemometric target fishing models are highly dependent on a reasonable sample of active conformations. Since a number of diverse conformational sampling algorithm exist, which exhaustively generate enough conformers, however model building methods relies on explicit number of common conformers.

**Results:**

In this work, we have attempted to make clustering algorithms, which could find reasonable number of representative conformer ensembles automatically with asymmetric dissimilarity matrix generated from openeye tool kit. RMSD was the important descriptor (variable) of each column of the *N* × *N* matrix considered as *N* variables describing the relationship (network) between the conformer (in a row) and the other *N* conformers. This approach used to evaluate the performance of the well-known clustering algorithms by comparison in terms of generating representative conformer ensembles and test them over different matrix transformation functions considering the stability. In the network, the representative conformer group could be resampled for four kinds of algorithms with implicit parameters. The directed dissimilarity matrix becomes the only input to the clustering algorithms.

**Conclusions:**

Dunn index, Davies–Bouldin index, Eta-squared values and omega-squared values were used to evaluate the clustering algorithms with respect to the compactness and the explanatory power. The evaluation includes the reduction (abstraction) rate of the data, correlation between the sizes of the population and the samples, the computational complexity and the memory usage as well. Every algorithm could find representative conformers automatically without any user intervention, and they reduced the data to 14–19% of the original values within 1.13 s per sample at the most. The clustering methods are simple and practical as they are fast and do not ask for any explicit parameters. RCDTC presented the maximum Dunn and omega-squared values of the four algorithms in addition to consistent reduction rate between the population size and the sample size. The performance of the clustering algorithms was consistent over different transformation functions. Moreover, the clustering method can also be applied to molecular dynamics sampling simulation results.

**Electronic supplementary material:**

The online version of this article (doi:10.1186/s13321-017-0208-0) contains supplementary material, which is available to authorized users.

## Background

Clustering algorithms used in a variety of situations, such as understanding virtual screening results [1], partitioning data sets into structurally homogeneous subsets for modeling [2, 3], and picking representative chemical structures from individual clusters [4–6]. The use of clustering algorithms to group similar conformations is the most appropriate data mining technique to distill the structural information from properties of an MD trajectory [7–10]. Therefore, the selection of representative conformers is valuable and very important in the 3D-QSAR model, pharmacophore model, protein–ligand docking [11], and Bayesian classification model from 3D fingerprints. Various conformation-generating algorithms are commonly used in commercially available programs and open source wares. The performance of such conformation generators have been evaluated by assessing the reproducibility of the X-ray bioactive conformer [12]. The existence of the bioactive conformer supports evaluation of correct conformation of the automatically selected conformers. However, if X-ray bioactive conformer information do not exist then the local minimum conformers or conformer ensembles with reasonable sizes were chosen to build the 3D models with a statistically desirable result [13, 14].

Currently, the development of omics, network pharmacology and systems biology has motivated the field of chemo-informatics to predict the targets, off-targets, and poly-pharmacology of interesting compounds using in silico methods. Among these in silico target inference methods, the chemocentric approach (ligand-based target fishing) requires a simple assumption for structurally similar molecules have similar biological activity [15]. In general, this approach has used 2D structures for the similarity calculation rather than 3D structures due to the computational burden. However, 2D similar compounds can make highly experienced medicinal chemists suggest similar targets but it have less probability to give novel pharmacological effects in comparison to 3D similarity compounds [16, 17]. Hence, the computationally intensive 3D similarity based target fishing is required. However, 3D similarity depends on the 3D conformation and 3D alignment. In contrast to 3D models of a specific target using bioactive conformers from X-ray such like our previous studies [18–20], the recent studies used a single low energy conformer or conformer ensembles under a specific algorithm [17, 21, 22] to acquire the 3D structure of a query molecule for target fishing. Some conformer ensemble under this program with a default size (e.g., 1 or 100), determined the similarity scores, which were able to change the first ranked target in target fishing. In this study, we have tried to investigate clustering methods to acquire reasonably small-sized conformer ensemble, which are representing conformational space of a drug to build 3D models with high coverage. When PDBs of targets are unavailable, this approach is one plausible solution to get robust 3D-QSARmodels. In particular, we tried to propose the best clustering method to acquire reasonable ensembles by comparing four different types of conventional algorithms: (1) a representative conformer k-means algorithm, (2) a hierarchical clustering with dynamic tree cut algorithm, (3) a linear kernel principal component analysis, and (4) a non-linear kernel principal component analysis. All four algorithms work based on relative distances, so they can easily be extended to multi-dimensional dissimilarity. We noted that the relative distances are directed dissimilarities between conformers. Since different matrix transformation functions could detect different patterns, the algorithms need to be tested over different metrics (transformation method) including admissible methods with respect to the stability [23]. All algorithms could be implemented in the process consisting of (1) conformer ensemble generation by omega [24–26], (2) shape based alignment by a Shape toolkit [27–30], (3) asymmetric RMSD (root mean square deviation) calculation (*N* × *N*) by the OEChem toolkit [31], and (4) a RMSD-based selection of representative conformers. The main contributions of this work are the next two. First contribution is to make it easy to adopt clustering algorithms for finding representative conformers with RMSD by automating the *k* and resolutions, which are required in the original clustering methods. The second is to provide the demonstration of the performance in finding representative conformers from initial sets with different clustering algorithms for reference information, so that researchers are able to find more proper algorithm for their research purposes.

## Methods

### RMSD matrix

**Fig. 1:**
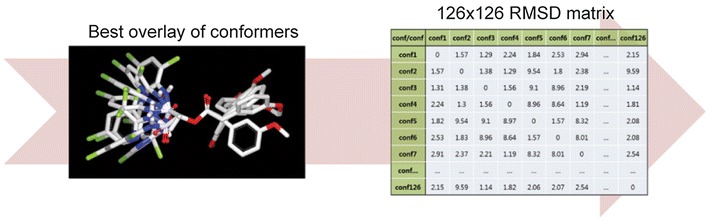
An example of aligned conformers and their RMSD matrix

Before describing the four automated resampling methods, the procedure to generate a conformer ensemble is illustrated. Shape-based alignments of the data sets in each conformer ensemble were conducted using OEChem [31] and the OEShape toolkit (OpenEye Scientific Software). All conformers were aligned based on the conditions of (1) brute forced *N(reference)* × *N(fit)* cases and (2) the class, “OEBestOverlay.” RMSD values between every aligned conformer were calculated to store these values in an *N* × *N* matrix, as shown in Fig. 1. In the *N* × *N* matrix, a row and a column are a conformer and a variable to use a total of *N* variables, even though RMSD was a variable to describe the relationship between a pair of conformers. The toolkit used for conformer generation, alignment of conformers, and RMSD calculation produced the non-symmetry matrix (but approximate symmetric) resulting from (1) selection algorithm of starting position for the alignment (inertial frame alignment algorithm), (2) rigidity of reference conformer during finding ‘centers-of-mass’, and (3) single selection from multiple OEBestOverlay results. Some dissimilarity values in RMSD were modified to make the RMSD matrix symmetric. The RMSD values generated by the toolkit have all positive values satisfying $d\left( {x,y} \right) \ge 0$. Some dissimilarity values in diagonal does not satisfy the property of $d\left( {x,y} \right) = 0 \quad {\text{if}}\,x = y$, and the non-zero diagonal values changed to zero. We assume the reasons of the occurrence of the non-zero diagonal values are similar to the reasons for the non-symmetricity of the matrix: the starting position, the rigidity of reference conformer, etc. Further, to make the non-symmetric matrix symmetric, we applied matrix transformations for clustering. For the clusters built from directed networks, stability issue rises. It needed to be confirmed whether networks that are close to each other result in dendrograms that are also close to each other for a given hierarchical clustering algorithm. Carlsson et al. [23] defined a clustering algorithm is stable if $d_{N} \left( {H\left( {N_{X} } \right), H\left( {N_{Y} } \right)} \right) \le d_{N} \left( {N_{X} , N_{Y} } \right)$ for all ${\text{N}}_{\text{X}} , {\text{N}}_{\text{Y}} \in {\text{N}}$. Carlsson et al. [23] proved reciprocal clustering and non-reciprocal clustering satisfies stability. Reciprocal clustering defines the cost of an edge as the maximum of the two directed dissimilarities. The matrix transformation for reciprocal clustering can be formulated as: $\bar{A}_{X} : = \hbox{max} \left( {A_{X} , A_{X}^{T} } \right)$, where the max is applied element-wise. And a transformation for non-reciprocal clustering can be defined as: $\bar{A}_{X} : = \hbox{min} \left( {A_{X} , A_{X}^{T} } \right)$. Other transformations could be lower-triangle, upper-triangle, and average that do not satisfy the stability. It is worth to build clusters with different transformations since we also needed to test whether one clustering algorithm performs higher than the others over similar variations of dataset. When conducted clustering from RMSD matrix, lower diagonal part of the matrix was used in this study. The upper triangle part is removed and replaced by the lower triangle part to gain a symmetric matrix. Our manipulation on the matrix means that real value ‘RMSD(A,B)≠RMSD(B,A)’ approximately assumed into ‘RMSD(A,B)=RMSD(B,A)’.

### Representative conformers and clusters

We define a representative conformer ensemble as a subset that can describe the total sets in the best way. Each conformer in the subset was expected to be dispersed and to belong to each sub-group in the total set if any. The similarity and distance between conformers can be calculated by relative distance (not by absolute distance). The error would become greater if used a medoid instead of a mean due to the difficulty of calculating an absolute distance [32]. One way to calculate the mean center points with a relative distance is to convert the relative distances from each point to absolute distances from some virtual local points (support vectors) [33]. Here, the whole conformers were used as support vectors because we did not want to lose information.

When use clustering algorithm we need to define a good cluster. Even though there does not exist a good definition for a good cluster that can be applied to every application domain [34], we follow a general definition—a cluster is a set of data objects that are similar to each other, while data objects in different clusters are different from one another [35]. However, we note that a good cluster in our research should explain diverse different characteristics of a dataset. Among recent reports on clustering for representative conformers, Kim et al. attempted to find representative conformers using divisive clustering methods from a large PubChem3D [36] conformer set [37]. Kothiwale et al. [38] used knowledge such as ‘rotamer’ libraries. Feher and Schmidt used the fuzzy c-means clustering method to find representative conformers using quantities and features inherent to the dataset [39].

### Automated resampling methods

Heuristic and approximation methods were applied to our clustering problem in this study because the clustering problem consider an NP (nondeterministic polynomial time) problem [40]. The four clustering methods are (1) the k-means clustering of multidimensional scaled RMSD values based on a linear kernel without suppling *k* explicitly, (2) the hierarchical clustering algorithm with dynamic tree cut based on a linear kernel without using an explicit threshold, (3) PCA (principal component analysis) with a linear kernel and (4) PCA with an RBF (radial basis function) kernel.

When using clustering for representative conformers, it is a limitation of this research that deterministic initial methods were not applied such as initializing *k* centroids far apart from each other [41–43], and adopting deterministic initialization [44–46]. In this research, the initial centroids randomly was set and the greatest result was chosen after multiple runs. It is a limitation that the k-means algorithm returns different representative conformers every running with respect to the deterministic representativeness of representative conformers. We propose the application of deterministic initial centroids to a k-means algorithm in detection of representative conformers as a future work. In this work, we attempted to increase the adaptability of k-means for representative conformer set by automating the option of *k*.

We also included hierarchical clustering and PCA based clustering for the comparison. When disable to estimate the shape of clusters in a conformer dataset in advance, a hierarchical clustering is a proper choice [47]. The clusters as a result become different depending on the resolution to the hierarchical tree. Since the resolution varies for each conformer dataset, it should be automated. To find linear characteristics of a conformer dataset, PCA is used for clustering.

#### k-Means clustering

The first trial performs to cluster the conformers and select representative conformers within the clusters. k-Means clustering using *n* variables acquired from multidimensional scaling of *N* dimensional variables in the matrix was performed to select representative conformers. k-Means is one of the most popular clustering methods, which tries to minimize the sum of the squared distance within the clusters [48]. However, k-means has a few disadvantages: it cannot find the global optimum and the user needs to specify the number of clusters, *k*. Our algorithm finds *k* automatically by aiming to maximize the descriptive power of the representative conformers based on MSQb. We expect descriptive representative conformers may minimize the mean of the squared distance of the clique within clusters (MSQw) and to maximize the mean of the squared distance of the clique between the clusters (MSQb). The conformers in a cluster would be similar to each other (like a clique) considering that the relative distances are based on the similarity among conformers. A clique is a group of conformers that were on average more similar to each other than any others.^1^ The representative conformers based on the clique can be formulated as:

1 $$max_{k} \frac{1}{{2*C\left( {k,2} \right)}}\mathop \sum \limits_{i = 1}^{k} \mathop \sum \limits_{j = 1, i \ne j}^{k} \left| {\left| { c_{i} - c_{j} } \right|} \right|^{2} ,$$

2 $${\text{s}}.{\text{t}}.\quad min\frac{1}{k}\sum\limits_{c \in G} {\frac{1}{{C\left( {c_{k} ,2} \right)}}\mathop \sum \limits_{i = 1}^{{c_{k} }} \mathop \sum \limits_{j = 1}^{{c_{k} }} C_{ij} \left| {\left| { x_{i} - x_{j} } \right|} \right|^{2} } ,$$

where the formula is MSQb (Eq. 1) and the constraint is MSQw (Eq. 2). The number of clusters is *k*; the representative conformers for each cluster are *c*_*i*_ and *c*_*j*_. The number of conformers for each cluster is *c*_*k*_. *C*_*ij*_ is an index matrix that denotes whether each conformer belongs to a cluster or not (consisting of 0 or 1). *C*(*c*_*k*_,2) is the number of possible combinatorial cases.

**Fig. 2:**
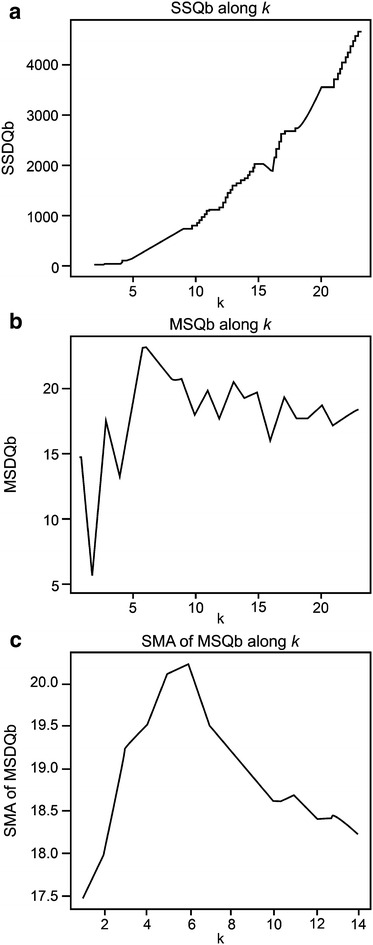
The trend of the squared distance of the clique between clusters for the entry 10; **a** SSQb along to *k*, **b** MSQb along to *k*, **c** SMA of MSQb along to *k*

In k-means clustering, the sum of the squared distance of a clique within a cluster (SSQw) declines as the number of clusters increases. The sum of the squared distance of a clique between clusters (SSQb) has a tendency to increase as the number of clusters increases, even though there were some variations in this trend (Fig. 2a). However, MSQb shows different patterns, where it stops increasing after a certain point (Fig. 2b). A simple moving average (SMA) was applied to smooth the MSQb curve. The example below used a window size (*W*) of 10. We used the highest point of MSQb as the number of clusters, *k* (Fig. 2c).

3 $$SMA = \frac{1}{\text{W}} \mathop \sum \limits_{{{\text{i}} = 0}}^{{{\text{W}} - 1}} {\text{MSQb}}_{{{\text{K}} - {\text{i}}}}$$

**Scheme 1:**
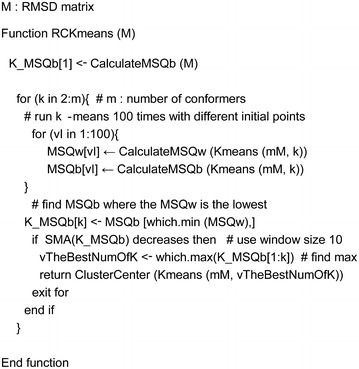
k-means algorithm for representative conformers (RCKmeans)

The algorithm using k-means to find the representative conformers was expressed as RCKmeans (representative conformer k-means) and is described in Scheme 1. Initially, we ran k-means 100 times with different initial points to find the lowest MSQw. Since k-means finds local optimums, it is necessary to reinforce the results with different initial points. Next, the algorithm repeated this step with increasing *k*. As *k* increased, the algorithm calculated the SMA with a window size of 10. When SMA started to decrease, RCKmeans tried to find the highest value for MSQb and returned the *k* at that time. Once the *k* clusters were detected, the conformers at the center of each cluster were selected as representative conformers the Cluster Center function did this.

#### Hierarchical clustering with dynamic tree cut

Hierarchical clustering is a bottom-up method, whereas k-means a divisive method. Hierarchical clustering techniques also popular for clustering. Hierarchical clustering requires a branch pruning procedure to make the clusters more meaningful with respect to the cluster sizes and the number of clusters. Langfelder et al. [47] tested different pruning methods and suggested the dynamic tree cut method for complex trees where one cannot find all of the clusters with one cut height (static method). The dynamic tree cut method starts to merge branches from the bottom to the top. The merging of two branches was evaluated by shape criteria. We used the minimum number of objects, the core scatter of the tree, and the gap between the branches as the shape criteria, as in [47].

**Scheme 2:**
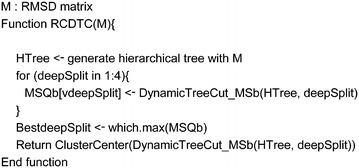
The DynamicTreeCut algorithm for representative conformers (RCDTC)

**Fig. 3:**
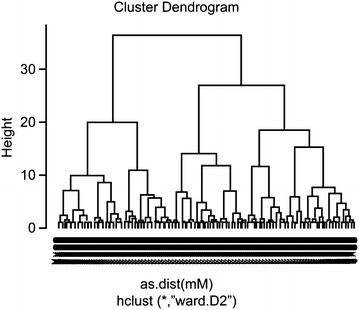
Example of a hierarchical tree and tree cut with the entry 29

Therefore, we adapted the dynamic tree cut method for clustering conformers in an entry. To remove the user’s explicit intervention of specifying the depth of the tree cut and separation, our pruning method tested four different depths and chose the depth where MSQb was the highest and the fewer in the sizes of clusters as described in Scheme 2 and Fig. 3. The tree was constructed based on the ward’s minimum variance distance (MSw: mean squared distance within). Ward’s method built trees in a way to minimize the variance [51, 52]. The DynamicTreeCut algorithm for the representative conforms (RCDTC) is implemented within R [47]. Conformers that do not belong to any clusters could remain when tree cut. These outsides were assigned to the nearest clusters by PAM (partitioning around medoids) stages. Once the clusters were identified, the conformers at the center of each cluster were selected as representative conformers-the Cluster Center functionalized.

#### Kernel PCA

PCA used in many applications (e.g., data compression, visualization). PCA represent the differences with k-means to find representative conformers, and provides different results from k-means. k-means finds the representative conformers by the shape of the distances between the center and closer elements. However, PCA tries to determine the orthogonal linear pattern first, and then finds representative conformers based on the linear pattern. In factor analysis, PCA identified the variables with stronger factor loadings [53]. PCA detected linear patterns and then considered the conformers with the strongest factor loadings as representative conformers. Kernel PCA [54] used linear or a nonlinear form of PCA, and an applicable method for finding various types of relations among conformers. The covariance matrix of the data, $x_{j} ,j = 1 \ldots , \, m,x_{j} \in R^{N}$, is defined as $C = \frac{1}{\text{m}}\sum\nolimits_{j = 1}^{m} {x_{j} x_{j}^{T} }$. While assuming that our conformer was mapped into feature space, $\varphi \left( {x_{1} } \right) \ldots ,\varphi \left( {x_{m} } \right)$, the covariance matrix for PCA is as follows:

4 $$\bar{C} = \frac{1}{\text{m}}\mathop \sum \limits_{j = 1}^{m} \varphi \left( {x_{j} } \right)\varphi \left( {x_{j} } \right)^{T}$$

We mapped a conformer into an infinite-dimensional feature space with the linear operator *φ*(*x*_*j*_)*φ*(*x*_*j*_)^*T*^ and calculated eigenvalues and eigenvectors. This way, we could calculate the distance between two conformers without knowing the absolute coordinates in 3D. To calculate the principal components of a test point *x*, we computed projections onto the eigenvectors, *V* ^*n*^. The mathematical detailed proof of the following formula can be found in the Ref. [55].

5 $$\left( {V^{n} ,{\varphi }\left( x \right)} \right) = \mathop \sum \limits_{i = 1}^{m} \alpha_{i}^{n} \left( {\varphi \left( {x_{i} } \right),\varphi \left( x \right)} \right) = \mathop \sum \limits_{i = 1}^{m} \alpha_{i}^{n} K\left( {x_{i} ,x} \right),\quad {\text{n}} = 1 \ldots ,{\text{p}}$$

where kernel $K\left( {x_{i} ,x} \right) = \left\{ {{\varphi }\left( {x_{i} } \right),{\varphi }\left( x \right)} \right\}$ can be calculated without the explicit definition of φ. The linear kernel is defined as:

6 $$K_{linear} \left( { x_{i} ,x} \right) = \left\| {x_{i} - x} \right\|^{2}$$

The values generated by the kernel function were analyzed using PCA, which could reasonably reduce the number of variables to produce components with minimal distortion of the data. At 80% explanatory power (in other words, the information loss was less than 0.2), the major component contributions were extracted among *N* variables:

7 $$\frac{{\frac{1}{m}\mathop \sum \nolimits_{i = 1}^{m} \left\| {\varphi \left( {x_{i} } \right) - \varphi (x_{i} )_{approx} } \right\|^{2} }}{{\frac{1}{m}\mathop \sum \nolimits_{i = 1}^{m} \left\| {\varphi \left( {x_{i} } \right)} \right\|^{2} }} \le 0.2$$

For example the eigenvector tables consisted of components (column) and conformers (row) as shown in Table 1. The second row represented cumulative explanatory power. From these components, the most representative conformers were chosen from the eigenvector tables. To choose the representative conformers, and kept the highest absolute values in each row and then chose the highest absolute values among the highest in each column. This way the most effective conformer chosen for each component. After limiting the explanation coverage to 80%, the four dimensions (V1–V4) chosen out of the 41 possible dimensions within the example. The values in italic font in the eigenvectors table became the representative conformers. This process knows as RCPCA (PCA for representative conformers).

**Table 1:** Identification of principal conformers by RCPCA with the entry 29 (an example) after limiting the explanation coverage to 80%

Conformer Id	Factor 1	Factor 2	Factor 3	Factor 4
(Cumulative explanatory power)	49.4%	66.0%	74.4%	81.7%
1	0.166	−0.107	−0.040	−0.012
…	…	…	…	…
16	0.246	−0.448	−*0.673*	0.396
17	−0.179	−0.028	−0.014	0.014
18	0.073	0.136	−0.156	−0.171
…	…	…	…	…
23	0.053	*0.467*	−0.236	0.032
24	0.173	0.004	0.081	0.084
25	0.166	0.008	0.033	0.034
…	…	…	…	…
34	−0.120	0.282	0.051	*0.433*
35	0.198	0.163	−0.012	−0.043
36	−0.002	0.010	0.287	0.271
37	−0.182	−0.068	−0.145	−0.087
38	*0.203*	0.063	0.021	−0.018
…	…	…	…	…

#### Nonlinear kernel PCA

Nonlinear patterns may describe the conformer set in a more suitable way. For nonlinear PCA, the RBF kernel can be used [54]. Consecutively, the selection of representative conformers by kernel PCA was conducted to minimize distortion of the raw data (RMSD matrix). The conversion of RMSD values by RBF kernel requires *σ* ^2^ as in Eq. 8. The *σ* ^2^ should be calculated separately for each entry. The standard deviation of an entry calculated by the relative distances. The whole number distance between two conformers is *C*(*m*,2), where *m* is the size of an entry. We considered the mean of the standard deviation among the conformers as the standard deviation of the entry. The parameter, *B*, was designed for a generalization purpose. When *B* was less than 1, the kernel PCA had a tendency to find patterns by using the conformers closer to the support vectors, and vice versa. This value set *B* to 1 by default. The PCA method with RBF kernel was named RCPCA_RBF. The RBF kernel is defined as follows:

8 $$K_{RBF} \left( { x_{i} ,x} \right) = { \exp }\left( {\frac{1}{B} \times \frac{{\left\| {x_{i} - x} \right\|^{2} }}{{2\sigma^{2} }}} \right)$$

The Wilson–Hilferty transformation was used to alleviate the skew caused in the higher dimension space [56]. The average (*E*) of the sum of squared distances takes the power of 1/3. The value of *σ* is calculated as follows:

9 $$\upsigma = \sqrt[3]{{{\text{E}}\left( {2 \left( {\frac{{\left\| {{\text{x}}_{\text{i}} - {\text{x}}} \right\|}}{2}} \right)^{2} } \right)}}$$

### Data set

#### Conformer set

In public database, 3D-conformations of the chosen chemicals were generated by omega after the removal of molecules with a hypervalent metal complex due to the assignment of charge under the Merck molecular force field (MMFF) [14, 37]. The energy window for conformer generation was selected based on the previous publications [4]. In the selection of the dataset for our study, the ideal criteria were: (1) the number of conformers (*N*) within a fixed energy window and (2) the difficulty of clear groupings in *N* by the *N* RMSD matrix. Our method should work well in all compounds; however, the results from examples with different rotatable bonds could confirm the algorithm performance. To be close to an ideal data set, structure diversity of our data set could be obtained through MACSS (structural key) based k-means clustering. In addition, the four properties also were considered for the selection of the data set; (2) NA (the number of heavy atoms), (3) NRB (the number of rotatable bonds), (4) NRE [nreffect = abs (NRB + (SR − SA)/5)]. In Table 2, 47 compounds with more than five rotatable bonds were selected using Knime [57].

**Table 2:** Properties of the entries

Entry	Compound ID	Structure (SMILES)	CS	Cluster no 1	Cluster no 2	NA	NRB	SBN	NRE	SR	SA
1	CHEMBL43055		0	5	17	15	4	4	4.8	4	0
2	CID 805065		–	13	22	23	6	0	6	9	9
3	CHEMBL1370046		9	31	27	30	7	5	8	11	6
4	CHEMBL1370087		9	35	10	28	5	9	6.8	15	6
5	CHEMBL1370039		9	28	2	24	6	0	6	9	9
6	CHEMBL1370041		9	7	49	25	7	0	7	6	6
7	CHEMBL1370046 (enantiomer of entry 3)		9	31	27	30	7	5	8	11	6
8	CHEMBL1370055		8	30	22	22	7	0	7	6	6
9	CHEMBL1370069		9	7	42	31	9	4	9.8	13	9
10	CHEMBL1370076		9	22	18	28	6	5	7	11	6
11	CHEMBL1370086		9	4	22	28	8	5	9	14	9
12	CHEMBL1428166		9	15	13	31	14	0	14	6	6
13	CHEMBL1801761		9	5	28	45	14	9	15.8	21	12
14	CHEMBL1418972		9	3	28	28	5	6	6.2	12	6
15	CHEMBL1418877		9	22	25	18	4	0	4	9	9
16	CHEMBL1807239		9	1	44	36	8	5	9	14	9
17	CHEMBL1808501		9	26	31	25	5	0	5	9	9
18	CHEMBL1419480		9	32	8	26	7	6	8.2	9	3
19	CHEMBL1419488		9	19	33	34	5	11	7.2	20	9
20	CHEMBL1813553		9	8	9	32	14	3	14.6	12	9
21	CHEMBL1814111		9	20	2	43	12	0	12	15	15
22	CHEMBL1419571		9	27	43	26	6	0	6	6	6
23	CHEMBL1419632		9	7	3	27	6	0	6	9	9
24	CHEMBL1419023		9	16	10	35	3	11	5.2	20	9
25	CHEMBL1829854		9	12	15	39	14	0	14	9	9
26	CHEMBL1829855		9	7	15	34	11	0	11	9	9
27	CHEMBL1834490		9	6	43	40	9	0	9	15	15
28	CHEMBL1418865		9	12	23	32	8	6	9.2	15	9
29	CHEMBL1864043		9	81	26	37	12	5	13	11	6
30	CHEMBL1420120		9	69	33	35	12	10	14	13	3
31	CHEMBL1419065		9	2	10	34	7	5	8	14	9
32	CHEMBL1876846		9	2	26	38	11	0	11	15	15
33	CHEMBL1420394		9	11	41	24	3	11	5.2	17	6
34	CHEMBL1419109		9	16	11	19	4	5	5	8	3
35	CHEMBL1420685		9	20	35	21	3	3	3.6	9	6
36	CHEMBL1420706		9	18	46	21	3	7	4.4	13	6
37	CHEMBL1420986		9	8	19	28	8	0	8	6	6
38	CHEMBL1421466		9	11	1	31	9	0	9	9	9
39	CHEMBL1419208		9	26	22	21	4	0	4	9	9
40	CHEMBL1421682		9	16	36	28	8	6	9.2	12	6
41	CHEMBL1421713		9	18	44	28	7	0	7	9	9
42	CHEMBL1421773		9	14	31	26	7	0	7	9	9
43	CHEMBL1422043		9	19	1	26	4	5	5	14	9
44	CHEMBL1422559		9	14	12	30	8	6	9.2	12	6
45	CHEMBL1422645		9	1	44	31	9	0	9	9	9
46	CHEMBL1422874		9	15	42	26	8	0	8	9	9
47	CHEMBL1424484		9	13	11	29	7	11	9.2	14	3

*CS* confidence score of bioassay, *Cluster no 1* the number of cluster after Kmeans clustering of compounds with identical rotatable bonds, *Cluster no 2* the number of cluster after Kmeans clustering of compounds chosen in first clustering. *NA* the number of heavy atoms, *NRB* the number of rotatable bonds, *SBN* the number of real single bonds in ring, *NRE* nreffect = abs (NRB + (SR − SA)/5), *SR* the number of apparent single bonds in ring, *SA* the number of apparent single bonds in aromatic ring

#### Evaluation criteria

To obtain ensembles of each representative statistical analysis of sampling method result was performed for the evaluation of the identified conformers. In statistics, if any sample is representative of a population, the sample can be called by a complete sample. A compete sample was used for inferences or extrapolations to the population. The statistical parameters (mean, standard deviation) of the samples from the four different clustering methods were calculated because they described the distribution of each sample under parametric statistics. In this study, eta-squared and omega-squared values were used to evaluate the explanation power of the algorithms, and the conventional evaluations indices are also applied, which are Dunn index and Davies–Bouldin index [58]. A clustering algorithm for representative conformer sets may be considered better than another if it surpasses the performance of another across various validity indices [59]. Dunn index [60] assigns greater values to sets of clusters that are compact and well-separated clusters with a small variance between members of the cluster. Since the Dunn index considers the distance between clusters and the size of clusters, the highest value indicates optimal number of clusters.

$${\text{Dunn index}} = \mathop {\hbox{min} }_{1 \le i \le n} \left\{ {\mathop {\hbox{min} }_{1 \le j \le n, i \ne j} \left\{ {\frac{{d\left( {i,j} \right)}}{{\mathop {\hbox{max} }\limits_{1 \le k \le n} d^{{\prime }} \left( k \right)}}} \right\}} \right\}$$

where, $d^{{\prime }} \left( k \right)$ stands for the distance in cluster *k*.

Davies–Bouldin index yields lower value for more quality clusters, so the lowest value with k indicates optimal number of clusters [61].

$${\text{Davies}} - {\text{Bouldin index}} = \frac{1}{k}\mathop \sum \limits_{i = 1}^{k} \mathop {\hbox{max} }\limits_{i \ne j} \left( {\frac{{\sigma_{i} + \sigma_{j} }}{{d\left( {c_{i} ,c_{j} } \right)}}} \right)$$

where, the $\sigma_{x}$ is the average distance between any data in cluster *x* and $c_{x}$. $d\left( {c_{i} ,c_{j} } \right)$ is the distance between two centers. Davies–Bouldin index evolved with different versions. We depict “complete” intra cluster distance and “single” inter cluster distance. When tested with “average” intra cluster distance, the results showed similar patterns in our experiment and we omit the illustration.

Eta-squared (η^2^), a nonparametric statistical method, defines how well the representative conformers explain the distribution [62]. A larger eta-squared value indicates a better representation of the distribution.

10 $$\eta^{2} = \frac{\text{SSb}}{\text{SSt}}$$

However, there are limitations of the bias and accuracy in eta-squared [63, 64]. To overcome these limitations, we also calculated omega-squared (*ω* ^2^). A greater omega-squared value indicates a better representation of the distribution [62].

11 $$\omega^{2} = \frac{{{\text{SSb}} - \left( {{\text{k}} - 1} \right){\text{MSw}}}}{{{\text{SSt}} + {\text{MSw}}}}$$

## Results and discussion

### Implications of the conformational space

**Fig. 4:**
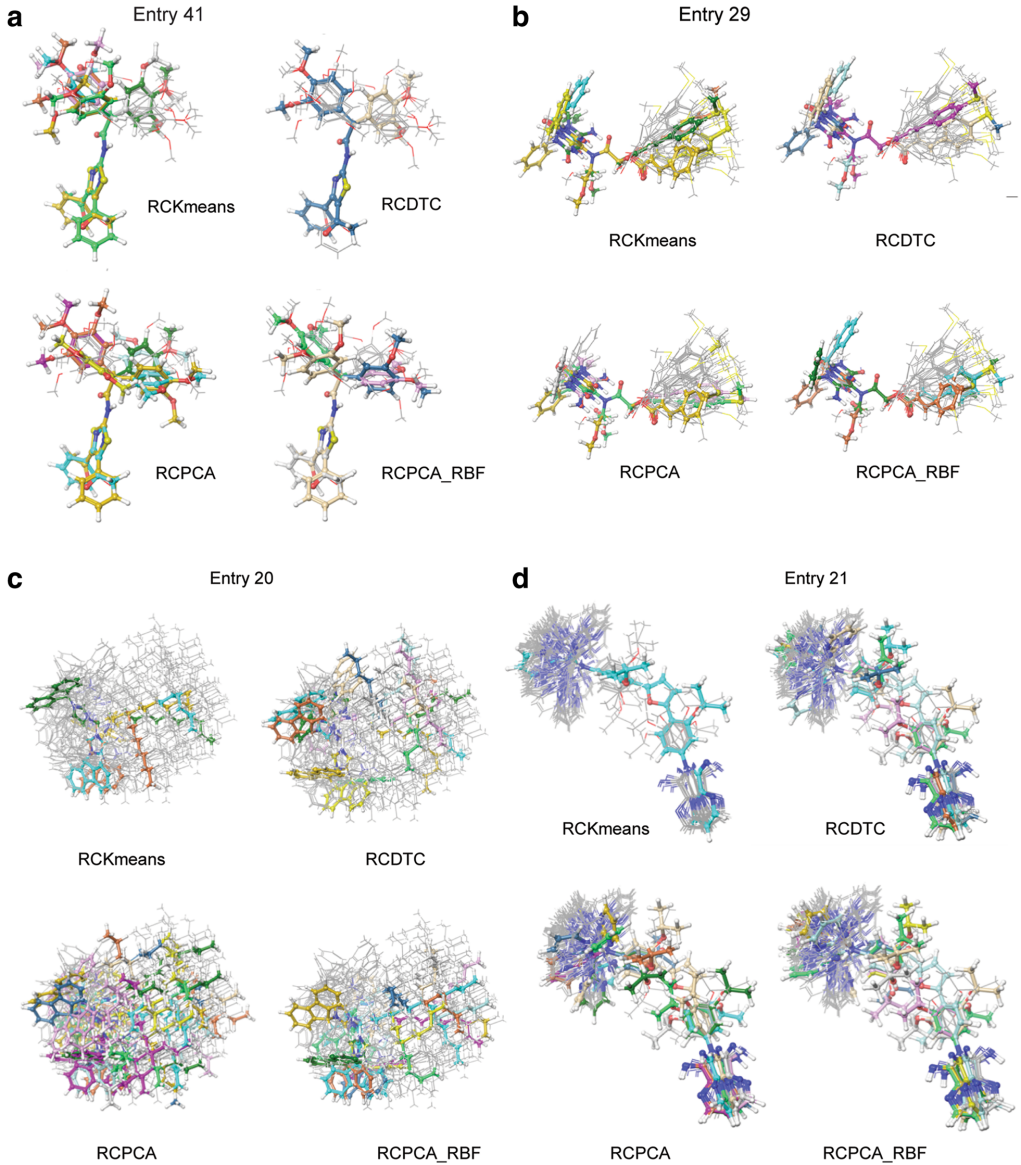
Conformations of the entries in 3D chemical space; **a** Entry 41, **b** Entry 29, **c** Entry20, **d** Entry 21; representative conformers are presented by ‘ball and stick’

Our main contribution is on investigating clustering algorithms to find the reduction (abstraction) rate of the data, correlation between the population and sample, explanatory power, the computational complexity, and the memory usage. For this purpose, we apply four different clustering methods. Table 3 presents the number of representative conformers according to each sampling method. The pattern of the sampling numbers was RCPCA ≫ RCPCA_RBF > RCDTC > RCKmeans. Some outliers from the general pattern could be observed in entry 9, 20, 21, 29, 41, etc. Entries 9 and 20 showed an excessive number of samples in RCPCA. While entry 21 showed only one representative conformer in RCKmeans, entries 29 and 41 showed that the number of the samples extracted from RCKmeans was the largest. Four entries were displayed in 3D chemical space (Fig. 4). Every conformation of the 47 entries in 3D chemical space is available in the supplementary information (Additional file 1: Fig. S1). When the representative conformers (ball and stick) and the other conformers (gray wires) were carefully observed, the representative conformers in Fig. 4 helped us to judge the coverage of the representative conformers in an entry. PCA presented the best coverage of all methods due to an excessive sample number. Only two conformers chosen from the dynamic tree cut could cover the variation of the 3,4-dimethoxyphenyl acetamide group in entry 41 (Fig. 4a).

**Table 3:** The number of representative conformer ensembles from four algorithms using lower triangle matrix

Entry	# of conf.	RCKmeans	RCDTC	RCPCA	RCPCA_RBF	Entry	# of conf.	RCKmeans	RCDTC	RCPCA	RCPCA_RBF
1	61	8	6	8	3	25	207	5	10	23	8
2	61	2	3	8	4	26	62	6	5	12	3
3	100	3	6	13	5	27	85	3	7	18	7
4	172	2	8	35	20	28	156	3	9	22	5
5	66	2	5	10	7	*29*	*41*	*5*	*4*	*4*	*3*
6	126	6	6	25	15	30	146	6	7	21	9
7	101	5	7	11	5	31	162	3	8	29	9
8	157	2	4	15	8	32	186	7	8	11	4
*9*	*309*	*3*	*10*	*49*	*23*	33	12	2	3	4	3
10	171	2	9	37	17	34	19	2	4	5	3
11	150	2	8	20	8	35	13	6	3	4	3
12	132	3	6	9	4	36	64	5	7	14	6
13	469	2	12	22	6	37	14	2	3	4	4
14	24	2	4	5	5	38	88	3	6	19	7
15	26	2	5	7	5	39	12	5	2	4	3
16	82	9	6	13	4	40	141	2	7	26	8
17	45	4	6	10	6	*41*	*36*	*7*	*2*	*7*	*5*
18	51	2	2	7	4	42	48	3	4	5	2
19	12	2	4	2	3	43	48	7	5	10	5
*20*	*500*	*4*	*14*	*66*	*13*	44	49	2	6	10	7
*21*	*238*	*1*	*10*	*11*	*13*	45	16	4	3	4	3
22	56	2	7	13	6	46	79	3	6	14	9
23	67	4	6	17	8	47	203	2	7	18	10
24	80	2	7	11	6	Average	108	4	6	15	7

Bold are outliers (entry 9, 20, 21, 29, 41) in the sampling pattern

**Fig. 5:**
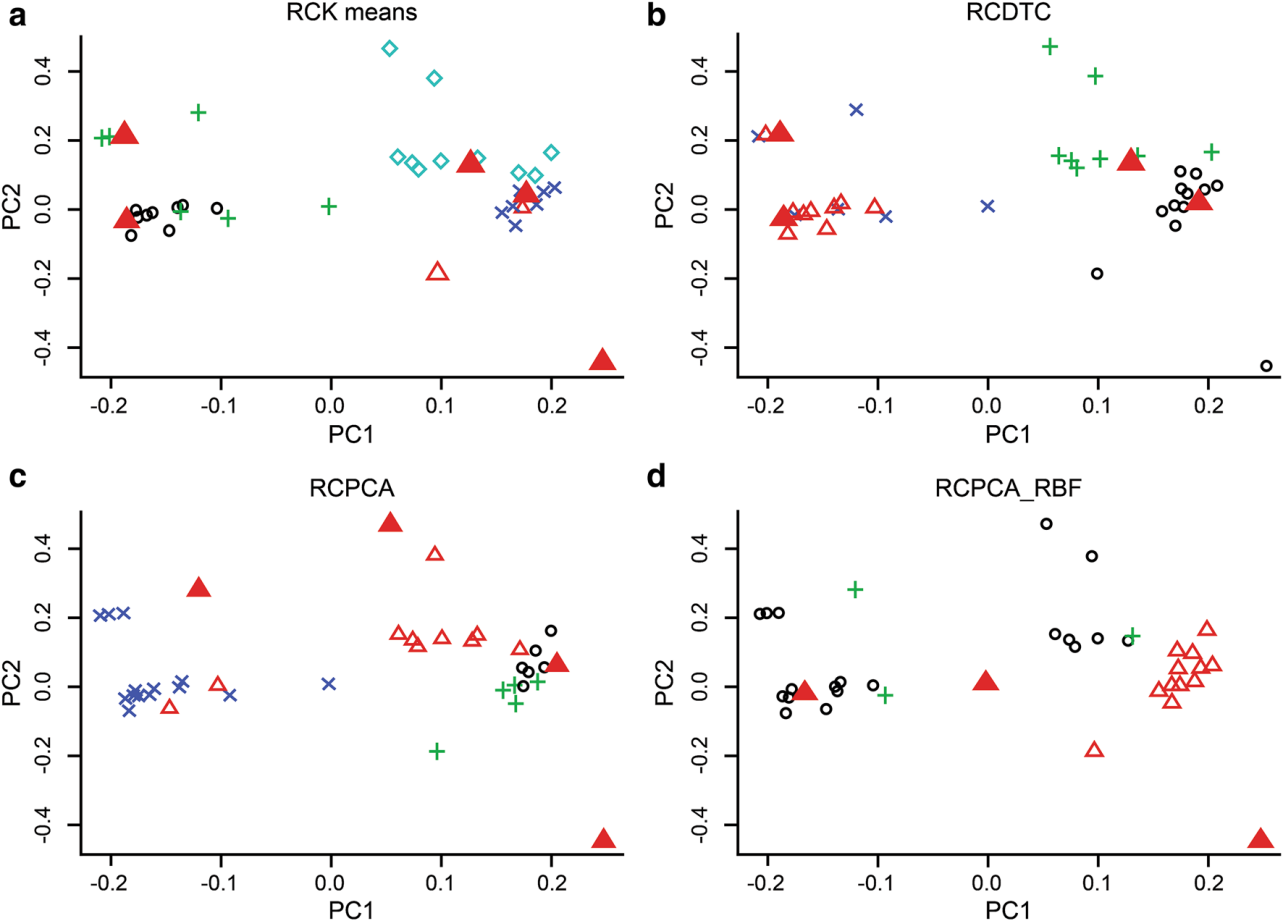
MDS (multi-dimension scaling) of an RMSD matrix and scatter plot of MDS-RMSD matrix. Clusters and representative conformers; **a** Entry 41, **b** Entry 29, **c** Entry20, **d** Entry 21

To visualize the conformers in a 2D scatter plot, the dimensions of the RMSD matrix were reduced using PCA. For example, the first and second principal components (PC1, PC2) from the 41 dimensions of the entry 29 were used for visualization in the Fig. 5. The conformers were presented with different colors and shapes according to their cluster. The representative conformers are marked with red triangles. The MSQb was the highest when the *k* number was 5, as shown in Fig. 5a. RCKmeans found five representative conformers. RCDTC, RCPCA, and RCPCA_RBF found 4, 4, and three of the representative conformers respectively. The five representative conformers of RCKmeans consisted of conformers 4, 5, 8, 10, and 16, and the four representative conformers of the dynamic tree cut consisted of 3, 4, 5, and 10 to show three consensus conformers. The four representative conformers of RCPCA consisted of 16, 23, 34, and 38, and three representative conformers of RCPCA_RBF consisted of 1, 16, and 36 to present conformer 16 as a common result. Conformer 4, 5, 10, and 16 were chosen in more than two methods and the overlap ones would be more reliable.

**Fig. 6:**
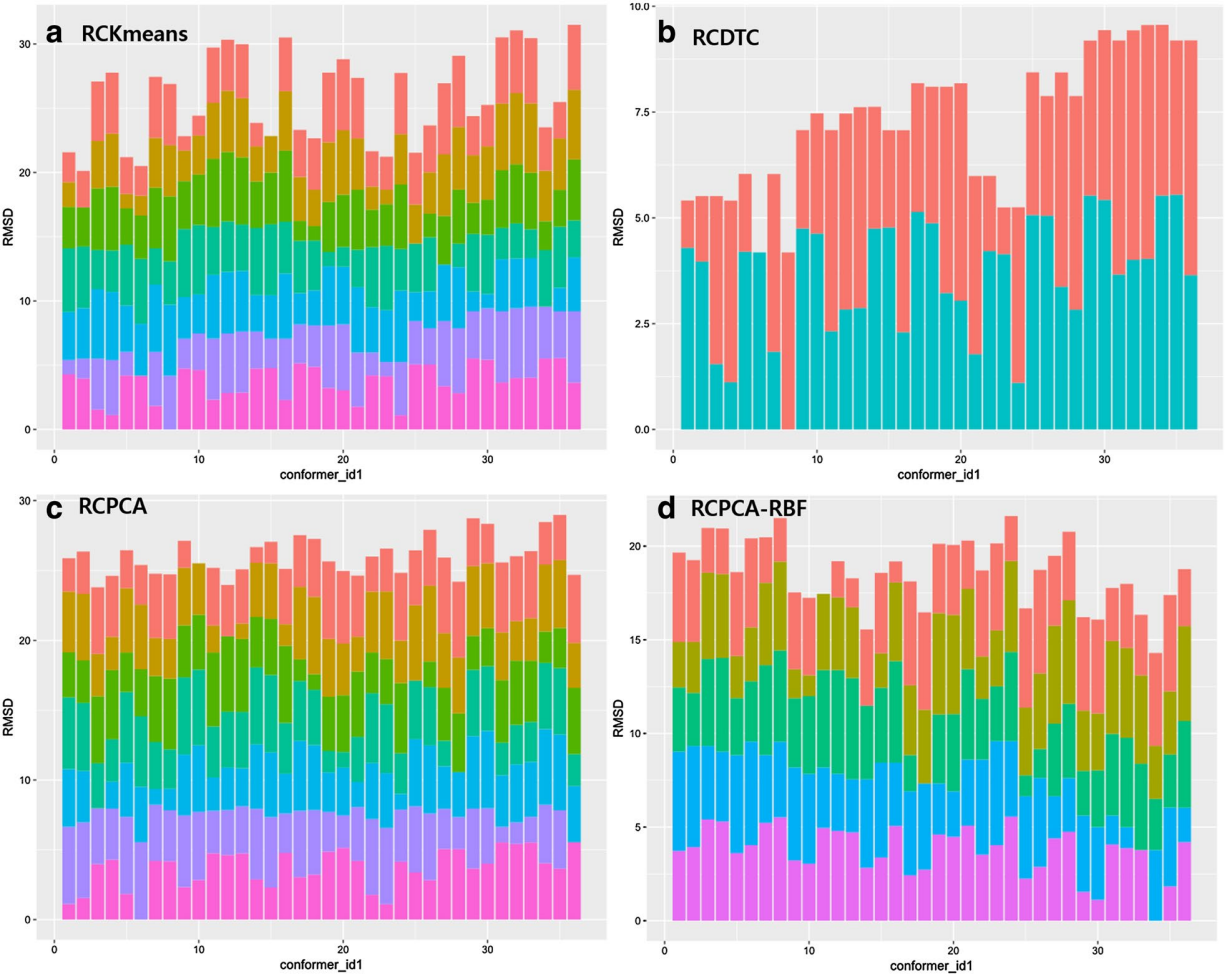
Histograms of the RMSD between four representative conformer ensembles and conformers of original set in the entry 41. **a** RCKmeans, **b** RCDCT, **c** RCPCA, and **d** RCPCA-RBF (*x*-axis: conformer ID in original set, *y*-axis: RMSD (Å), and *different colors* means each representative conformer ID chosen under the clustering)

In entry 29, the conformational variations were generated from (1) the *N*-benzyl group, (2) the *N*-methoxy ethyl group, and (3) the 3-(4-methylthio)phenyl acryloyl group (Fig. 4b). Among the three variations, the variation of the aryl acryloyl group occupied the largest space. Conformers 4, 5, 10, and 16 perfectly covered the space of the *N*-benzyl group without overlapping each other and a significant portion of the (4-methylthio)phenyl acryloyl group. Figure 6 represents the x-axis was the conformer number (total of 36 conformers) and the y-axis was the RMSD. Each line and color represents each representative conformer ensembles. The more the two lines are fare away each other means the more the two lines cover the conformational space.

### Structural characteristics of the representative conformer ensembles

During structural characteristic evaluation four representative conformers ensembles were found from 47 entries. The distributions of the conformers, the relations between the representative conformer ensembles and the whole conformers were analyzed to understand the characteristic of the algorithms. First, examined the distribution of the number of representative conformer ensembles of 47 data sets consisted of 107 conformers and result showed a large standard deviation (Table 4). The representative conformer ensembles were reduced to 19–14% of the initial size. RCKmeans chose the smallest number of representative conformers on average (3.58) and the lowest standard deviation (1.93). The number of representative conformer ensembles from RCDTC was similar to the one from RCPCA_RBF. These results indicated that if one reduced the standard deviation in the number of representative conformer ensembles, RCDTC would be more proper than RCPCA_RBF. However, we note that a greater number of representative conformer ensembles had a greater tendency for a bigger explanatory power, and vice versa.

**Table 4:** Mean number of representative conformers (R.C.) and correlation between No. of conformers and No. of R.C

Transformation methods	Clustering algo.	Number of R.C mean (SD)	Correlation (the number of conformers and the number of R.C)
Lower triangle	RCKmeans	3.58 (1.93)	0.11
	RCDTC	6.04 (2.61)	*0.87*
	RCPCA	14.88 (12.37)	0.8
	RCPCA_RBF	6.79 (4.53)	0.58
Upper triangle	RCKmeans	3.65 (2.52)	0.16
	RCDTC	5.92 (2.45)	*0.83*
	RCPCA	14.88 (12.37)	0.8
	RCPCA_RBF	6.79 (4.61)	0.58
Average	RCKmeans	3.88 (2.72)	0.07
	RCDTC	6.08 (2.61)	*0.81*
	RCPCA	13.98 (11.17)	0.75
	RCPCA_RBF	6.79 (4.52)	0.58
Reciprocal	RCKmeans	3.96 (2.96)	0.03
	RCDTC	5.94 (2.42)	*0.81*
	RCPCA	14.94 (12.54)	0.81
	RCPCA_RBF	6.83 (4.61)	0.57
	RCKmeans	3.69 (2.11)	0.11
	RCDTC	6.17 (2.66)	*0.89*
	RCPCA	14.56 (11.81)	0.78
	RCPCA_RBF	6.73 (4.39)	0.59

Next, analyzed the relation between the number of representative conformer ensembles and the number of elements in an entry. The entry sizes varied from 12 to 500. RCDTC had the greatest value (0.87) for the correlation value between the two numbers. This indicates that RCDTC found a greater number of representative conformer ensembles as the size of an entries increased. RCKmeans had a correlation value of 0.11, which indicated weak relations between the representative conformer ensembles and the elements in an entry. Another characteristic to consider when choosing a clustering method is the reproducibility. RCKmeans used random initial points for clustering. When repeated, the chances to find the same representative conformers as before would not be guaranteed. RCKmeans is not reproducible but the other clustering algorithms are reproducible.

During this study, we noted that instead of interpreting the strength of correlation as an evaluation indicator, it would be better to consider it as different characteristics that depend on the applications. If one wants an equal number of representative conformer ensembles independent of the size of an entry, the clustering method with a low correlation and low standard deviation would be the proper choice. Each of the four algorithms showed different characteristics from one another, providing chances to choose a proper algorithm with respect to the application domain. Different matrix transformation methods build different dissimilarity matrices, and the number of representative conformers became different depending on them. Even though there were small variances in the number of conformers, RCPCA consistently generated more number of representative conformers than RCPCA_RBF, RCDTC and RCKmeans (Table 4). For the correlation between the size of an entry and the number of representative conformers, RCDTC was best among all transformation methods.

### Explanatory power of representative conformers

We compared the clustering performance and the explanatory power of four algorithms in conformer dataset. In Table 5, the first and second columns showed the transformation methods and clustering algorithms, the third through sixth columns show the mean and standard deviation of Dunn index, Davies-Bouldin index, eta-squared and omega-squared values for the 47 entries. The correlation between the mean squared distances of the representative conformer and the whole conformers, shown in the seventh column.

**Table 5:** The comparison of the performance of the four clustering algorithms over different matrix transformation methods by various indices (Dunn, Davies–Bouldin, eta-squared and omega-squared) and correlations between MSt and MSb of clusters

Transformation methods	Clustering algo.	Dunn mean (SD)	Davies-Bouldin mean (SD)	Eta-squared mean (SD)	Omega-squared mean (SD)	Cor (MSt, MSb)
Lower triangle	RCKmeans	0.2 (0.24)	8.06 (4.55)	0.32 (0.18)	0.28 (0.16)	0.82
	RCDTC	*0.23* (0.24)	*6.73* (3.63)	0.4 (0.14)	*0.35* (0.14)	*0.9*
	RCPCA	0.18 (0.15)	7.22 (4.53)	0.45 (0.09)	0.33 (0.12)	0.83
	RCPCA_RBF	0.14 (0.12)	8.84 (5.33)	0.32 (0.11)	0.26 (0.12)	0.78
Upper triangle	RCKmeans	0.17 (0.29)	8.06 (4.55)	0.32 (0.18)	0.27 (0.17)	0.82
	RCDTC	*0.21* (0.24)	*6.58* (3.2)	0.39 (0.14)	*0.34* (0.15)	*0.89*
	RCPCA	0.18 (0.18)	7.02 (3.46)	0.45 (0.08)	0.34 (0.11)	0.82
	RCPCA_RBF	0.14 (0.14)	8.66 (5.02)	0.33 (0.1)	0.26 (0.11)	0.78
Average	RCKmeans	0.2 (0.22)	8.31 (5.32)	0.34 (0.18)	0.29 (0.16)	0.82
	RCDTC	*0.23* (0.24)	*6.61* (3.26)	0.41 (0.14)	*0.35* (0.15)	*0.87*
	RCPCA	0.17 (0.13)	7.24 (4.02)	0.44 (0.09)	0.33 (0.12)	0.81
	RCPCA_RBF	0.14 (0.12)	8.54 (4.64)	0.33 (0.11)	0.27 (0.11)	0.78
Reciprocal	RCKmeans	0.21 (0.24)	7.5 (3.57)	0.33 (0.18)	0.29 (0.17)	0.82
	RCDTC	*0.22* (0.24)	*6.8* (3.51)	0.4 (0.14)	*0.35* (0.15)	*0.91*
	RCPCA	0.17 (0.13)	6.83 (3.8)	0.45 (0.08)	0.34 (0.11)	0.82
	RCPCA_RBF	0.14 (0.11)	8.74 (5.07)	0.32 (0.11)	0.26 (0.11)	0.79
Non-reciprocal	RCKmeans	0.2 (0.22)	7.8 (4.05)	0.33 (0.17)	0.29 (0.16)	0.81
	RCDTC	*0.23* (0.24)	*6.68* (3.29)	0.4 (0.14)	0.35 (0.15)	*0.91*
	RCPCA	0.19 (0.15)	6.87 (3.57)	0.46 (0.07)	0.35 (0.11)	0.81
	RCPCA_RBF	0.14 (0.12)	8.96 (5.22)	0.32 (0.11)	0.26 (0.12)	0.78

Dunn index showed the greatest with RCDTC over five transformation methods, and Davies–Bouldin index was the lowest with RCDTC over other clustering methods as well. RCDTC showed the highest performance in these two conventional indices over other clustering algorithms. The eta-squared that represented the explanatory power was the lowest with RCPCA, however RCDTC provided the greatest omega-squared value of 0.35 after removing the overestimates. The omega-squared value of RCPCA became even lower than the average of all values (0.12).

We conducted a paired *t* test to see the statistical significance of the difference of the means for each of the 47 entries that had values from the four algorithms. RCDTC had the greatest omega-squared value, compared to other three algorithms. The omega-squared values from RCDTC was significantly higher than that from RCKmeans (p = 0.0003) and RCPCA_RBF (p = 0.000), with the exception of RCPCA (p = 0.337). MSt (the mean of the squared distance of total conformers) indicates how the conformers in an entry were dispersed and MSb does that for the representative conformer ensembles. If the correlation between MSt and MSb is high, we could predict that the whole conformers and the representative conformer ensembles have strong dispersion relations. RCDTC had the greatest correlation of 0.9 and was followed by RCPCA (0.83), RCKmeans (0.82), and RCPCA_RBF (0.78). RCDTC had the greatest correlation consistently over different transformation methods. The consistency of the performance order indicated that the difference of $d\left( {x,y} \right) \ne d\left( {y,x} \right)$ in RMSD matrix was not as significant as to affect the performance order of the algorithms.

### Computational complexity

The complexity of clustering algorithms is strongly related to the number *n* of data objects and the number *k* of clusters. From all experiments, the running times of four algorithms averaging 30 trials were compared. The run time shown in Table 6 is the sum of the running time (in s) for the 47 entries. RCPCA finished in 1.81 s, RCDTC (which had the greatest explanatory power) took 9.35 s. Cor (data size, run time) provided the relation between the size of an entry and the running time. RCDTC had the strongest correlation (0.99). The minimum running times were close to 0 for RCPCA and RCPCA_RBF, due to the small size of an entry, the smallest entry consisted of 12 conformers. The maximum running time was less than 3 s with RCDTC, RCPCA, and RCPCA_RBF. The maximum running time of RCDTC was 1.13 s (standard deviation = 0.06), which suggested the availability of using an online search.

**Table 6:** Run time comparisons of clustering algorithms

Clustering algorithms	Run time of all (s)		Cor (data size, run time)		Minimum run time		Maximum run time		Computational complexity	Memory usage
	Mean	SD	Mean	SD	Mean	SD	Mean	SD		
RCKmeans	602.61	19.22	0.35	0.01	0.81	0.03	45.49	1.36	${\text{O}}\left( {tkn} \right)$	${\text{O}}\left( {k + n} \right)$
RCDTC	9.35	0.51	0.99	0	0.02	0	1.13	0.06	${\text{O}}\left( {tn^{2} } \right)$	${\text{O}}(tn^{2}$)
RCPCA	1.81	0.11	0.88	0.01	0	0	0.58	0.03	${\text{O}}\left( {tn^{2} } \right)$	${\text{O}}\left( {{\text{n}}^{2} } \right)$
RCPCA_RBF	1.83	0.13	0.88	0	0	0	0.56	0.03	${\text{O}}\left( {tn^{2} } \right)$	${\text{O}}\left( {{\text{n}}^{2} } \right)$

The computational complexity of k-means can be O(*kn*) [58]. The complexity of RCKmeans became O(*tkn*) as it repeated *t* times until finding the peak point with increasing *k* (*n* is the number of conformers in an entry and *k* is the number of clusters). After applied computational complexity algorithm, RCDTC used a general agglomerative hierarchical clustering algorithm during building the tree. The complexity of an agglomerative hierarchical clustering algorithm became different depending on the distance function [65]. The complexity of RCDTC with Ward’s method was O(*n* ^2^). PCA used a singular value decomposition, which took O(*kn*) time [66]. The time complexity of RCPCA_RBF was similar to RCPCA.

Several works explore the relative accuracy of various clustering algorithms in extracting the right number of clusters from generated data. The algorithm kept only the representative conformer ensembles as results, and the memory usage followed the regular clustering algorithms. The memory usage increased as follows: k-means < hierarchical clustering algorithm < PCA (Table 6) [40, 66, 67]. In order to compare the actual running time, four algorithms were implemented in R 3.2.2 [29] and ran in the environment of Windows 10 OS, 16 GB RAM, and an Intel Core i5-5200 CPU (2.2 GHz). In the future, these algorithms could be implemented as a service system. Thus, a user could install Python [68] and R [69] and submit a run command with the input structure file (e.g., sdf, mol2, oeb), and the system would provide the structure files of the selected representative conformers.

## Conclusions

The work we present here analyzes and combines clustering partitions using four representative conformers ensembles were found from 47 entries as examples. This study intended to propose the representative conformers (with reasonable size) from conformational space because the automated conventional clustering methods did not require a learning process for determining the parameters or coefficients (as for conventional linear regression models). RCKmeans calculated the MSQb with increasing values of k, and then stopped after finding the maximum of MSQb. The second clustering method, RCDT performed with four different depths in a bottom-up hierarchical clustering selected the depth showing highest MSQb value. RCPCA and RCPCA_RBF extracted representative conformers at an explanatory power of 80%. All of the clustering methods are simple because they do not require any explicit parameters from the user; the algorithm automatically calculates all parameters and intends to maximize the explanatory power of the representative conformers. RCDTC was the most desirable clustering method presenting a consistent reduction of the data, the small size of a sample, and a high coverage of conformational space. In particular, if a drug has a long acyclic substituent (with high flexibility), the coverage of RCDTC (with less than half number of RC in RCPCA) was superior to the coverage of RCPCA. If a drug has the number of conformers less than 80 due to limited flexibility, RCDTC showed the least failure in acquiring 10% sized RC from original conformers. Even though RCDTC didn’t present the best mean of eta-squared, it provided the best mean values of omega-squared after the removal of the overestimate. The result could be supported by a paired *t* test between the omega-squared value of RCDTC and the other clustering methods. The paired *t* test proved the significant of difference between RCDTC and RCPCA_RBF, RCDTC and Kmeans. The paired *t* test with RCPCA not shown any significance but the average number of samples in the RCPCA was 2.5 times greater than RCDTC. In addition, this tendency for RCDTC was supported by a 3D picture of the representative conformers and histograms of RMSD between the representative conformers and the whole conformers in the entry.

Although this study used omega to generate the conformers, the performance of the clustering method was also retained for sampling conformers from the molecular dynamics simulation. The locally optimal sets of clusters for RCKmeans found by multiple retrials become different upon trials, so deterministic initialization methods need to be considered as a future work. The sequence process could add an advantage to the reported conformer sampling methods. The significance of this study is applicable to find plausible biological targets of new druggable scaffolds synthesized by chemical intuition without any biological background in future.

## Additional file

**Additional file 1.** Conformations of every entry under RCKmeans, RCDCT, RCPCA, and RCPCA-RBF.

## Abbreviations

MD: molecular dynamics
MDS: multidimensional scaling
MMFF: Merck molecular force field
MSb: mean of the squared distance between
MSw: mean of the squared distance within
MSQb: mean of the squared distance of the clique between clusters
MSQw: mean of the squared distance of the clique within clusters
PAM: partitioning around medoids
PCA: principal component analysis
RBF: radial basis function
RCDTC: DynamicTreeCut algorithm for the representative conformers
RCKmeans: representative conformer k-means
RCPCA: PCA for representative conformers
RMSD: root mean square deviation
SMA: simple moving average
SSQb: sum of the squared distance of a clique between clusters
SSQw: sum of the squared distance of a clique within a cluster

## Footnotes

1 In social science, a clique is a group of people who interact with each other more regularly and intensely than others in the same setting [49]. In graph theory, a clique is subset of vertices of an undirected graph, such that its induced subgraph is complete; that is, every two distinct vertices in the clique are adjacent [50].

## References

[1] Quevedo CV, De Paris R, Ruiz DD, Norberto de Souza O (2014). A strategic solution to optimize molecular docking simulations using fully-flexible receptor models. Expert Syst Appl.

[2] Li Y (2006). Bayesian model based clustering analysis: application to a molecular dynamics trajectory of the HIV-1 integrase catalytic core. J Chem Inf Model.

[3] Phillips JL, Colvin ME, Newsam S (2011). Validating clustering of molecular dynamics simulations using polymer models. BMC Bioinform..

[4] Landon MR, Amaro RE, Baron R, Ngan CH, Ozonoff D, McCammon JA (2008). Novel druggable hot spots in avian influenza neuraminidase h5n1 revealed by computational solvent mapping of a reduced and representative receptor ensemble. Chem Biol Drug Des.

[5] Deng J, Lee KW, Sanchez T, Cui M, Neamati N, Briggs JM (2005). Dynamic receptor-based pharmacophore model development and its application in designing novel hiv-1 integrase inhibitors. J Med Chem.

[6] Chen JY, Lonardi S (1992). Biological data mining.

[7] Shao J, Tanner SW, Thompson N, Cheatham TE (2007). Clustering molecular dynamics trajectories: 1.characterizing the performance of different clustering algorithms. J Chem Theory Comput.

[8] Torda AE, van Gunsteren WF (1994). Algorithms for clustering molecular dynamics configurations. J Comput Chem.

[9] Hartigan JA, Wong MA (1979). A k-means clustering algorithm. J R Stat Soc Ser C Appl Stat.

[10] De Paris R, Quevedo CV, Ruiz DD, Norberto de Souza O (2015). An effective approach for clustering InhA molecular dynamics trajectory using substrate-binding cavity features. PLoS ONE.

[11] Shim J, MacKerell AD (2011). Computational ligand-based rational design: role of conformational sampling and force fields in model development. Med Chem Commun..

[12] Agrafiotis DK, Gibbs AC, Zhu F, Izrailev S, Martin E (2007). Conformational sampling of bioactive molecules: a comparative study. J Chem Inf Model.

[13] Perola E, Charifson PS (2004). Conformational analysis of drug-like molecules bound to proteins: an extensive study of ligand reorganization upon binding. J Med Chem.

[14] Bolton EE, Kim S, Bryant SH (2011). PubChem3D: conformer generation. J Cheminform.

[15] Martin Yvonne C, Kofron James L, Traphagen Linda M (2002). Do structurally similar molecules have similar biological activity?. J Med Chem.

[16] Yera ER, Cleves AE, Jain AN (2011). Chemical structural novelty: on-targets and off-targets. J Med Chem.

[17] Nettles JH, Jenkins JL, Bender A, Deng Z, Davies JW, Glick M (2006). Bridging chemical and biological space: “target fishing” using 2D and 3D molecular descriptors. J Med Chem.

[18] Gadhe CG, Lee E, Kim MH (2015). Finding new scaffolds of JAK3 inhibitors in public database: 3D-QSAR models and shape-based screening. Arch Pharm Res.

[19] Kim MH, Ryu JS, Hah JM (2013). 3D-QSAR studies of 1,2-diaryl-1H-benzimidazole derivatives as JNK3 inhibitors with protective effects in neuronal cells. Bioorg Med Chem Lett.

[20] Kim MH, Chung JY, Ryu JS, Hah JM (2011). Structure tuning of pyrazolylpyrrole derivatives as ERK inhibitors utilizing dual tools; 3D-QSAR and side-chain hopping. Bioorg Med Chem Lett.

[21] AbdulHameed MDM, Chaudhury S, Singh N, Sun H, Wallqvist A, Tawa GJ (2012). Exploring polypharmacology using a ROCS-based target fishing approach. J Chem Inf Model.

[22] Liu XF, Ouyang SS, Yu BA, Liu YB, Huang K, Gong JY, Zheng SY, Li ZH, Li HL, Jiang HL (2010). PharmMapper server: a web server for potential drug target identification using pharmacophore mapping approach. Nucleic Acids Res.

[23] Carlsson G, Memoli F, Ribeiro A, Segarra S (2013) Axiomatic construction of hierarchical clustering in asymmetric networks. In: IEEE international conference on speech and signal processing (ICASSP), pp 5219–5223

[24] OMEGA (2015) OpenEye scientific software (ver. 2.4.6), Santa Fe, NM. USA. http://www.eyesopen.com

[25] Hawkins PCD, Skillman AG, Warren GL, Ellingson BA, Stahl MT (2010). Conformer generation with OMEGA: algorithm and validation using high quality structures from the Protein Databank and Cambridge structural database. J Chem Inf Model.

[26] Hawkins PCD, Nicholls A (2012). Conformer generation with OMEGA: learning from the data set and the analysis of failures. J Chem Inf Model.

[27] Shape TK (2015) OpenEye scientific software (ver. 1.9.3), Santa Fe, NM. USA. http://www.eyesopen.com

[28] Hawkins PCD, Skillman AG, Nicholls A (2007). Comparison of shape-matching and docking as virtual screening tools. J Med Chem.

[29] Haigh JA, Pickup BT, Grant JA, Nicholls A (2005). Small molecule shape-fingerprints. J Chem Inf Model.

[30] Boström J, Berggren K, Elebring T, Greasley PJ, Wilstermann M (2007). Scaffold hopping, synthesis and structure-activity relationships of 5,6-diaryl-pyrazine-2-amide derivatives: a novel series of CB1 receptor antagonists. Bioorg Med Chem.

[31] OEChem (2015) OpenEye scientific software (ver. 2.0.0), Santa Fe, NM. USA. http://www.eyesopen.com

[32] Maritz JS, Jarrett RG (1978). A note on estimating the variance of the sample median. J Am Stat Assoc.

[33] Schölkopf B, Smola A (2002). Learning with kernels.

[34] Jain A, Murty M, Flynn P (1999). Data clustering: a review. ACM Comput Surv.

[35] Xu R, Wunsch DC (2010). Clustering algorithms in biomedical research: a review. IEEE Rev Biomed Eng.

[36] PubChem3D Thematic Series. 2016. http://www.jcheminf.com/series/pubchem3d

[37] Kim S, Bolton E, Bryant S (2013) PubChem3D: conformer ensemble accuracy. J Cheminform 5(1). doi:10.1186/1758-2946-5-1 10.1186/1758-2946-5-1 PMC3547714 23289532

[38] Kothiwale S, Mendenhall JL, Meiler J (2015) BCL::Conf: small molecule conformational sampling using a knowledge based rotamer library. J Cheminform 7(1):47. doi:10.1186/s13321-015-0095-1 10.1186/s13321-015-0095-1 PMC4607025 26473018

[39] Feher M, Schmidt JM (2003). Fuzzy clustering as a means of selecting representative conformers and molecular alignments. J Chem Inf Comput Sci.

[40] Shindler M, Wong A, Meyerson AW (2011). Fast and accurate k-means for large datasets. In Adv Neural Inf Process Syst.

[41] Bahmani B, Moseley B, Vattani A, Kumar R, Vassilvitskii S (2012) Scalable kmeans ++. In: Proceedings of 38th international conference on very large data bases (VLDB)

[42] Arthur D, Vassilvitskii S (2007) k-means ++: the advantages of careful seeding. In: Proceedings of the eighteenth annual ACM-SIAM symposium on discrete algorithms (SODA)

[43] Katsavounidis I, Kuo CCJ, Zhang Z (1994). A new initialization technique for generalized Lloyd iteration. IEEE Signal Process Lett.

[44] Celebi ME, Kingravi H (2012) Deterministic initialization of the K-Means algorithm using hierarchical clustering. J Pattern Recognit Artif Intell 26(7). doi:10.1142/S0218001412500188

[45] Su T, Dy JG (2007). In search of deterministic methods for initializing kmeans and Gaussian mixture clustering. Intell Data Anal.

[46] Boley D (1998). Principal direction divisive partitioning. Data Min Knowl Disc.

[47] Langfelder P, Zhang B, Horvath S (2008). Defining clusters from a hierarchical cluster tree: the dynamic tree cut package for R. Bioinformatics.

[48] Lloyd SP (1982). Least squares quantization in PCM. IEEE Trans Inf Theory.

[49] Salkind N (2008). “Cliques” Encyclopedia of educational psychology.

[50] Alba RD (1973). A graph-theoretic definition of a sociometric clique. J Math Sociol.

[51] Ward JH (1963). Hierarchical grouping to optimize an objective function. J Am Stat Assoc.

[52] Varina T, Bureaua R, Muellerb C, Willett P (2009). Clustering files of chemical structures using the Székely–Rizzo generalization of Ward’s method. J Mol Graph Model.

[53] Malinowski ER (2002). Factor analysis in chemistry.

[54] Schölkopf B, Smola A, Müller K (2005). Kernel principal component analysis (Lecture Notes in Computer Science). Artif Neural Netw.

[55] Wilson E, Hilerty M (1931). The distribution of Chi square. Proc Natl Acad Sci.

[56] Terrell GR (2003). The Wilson–Hilferty transformation is locally saddle point. Biometrika.

[57] KNIME (2015) KNIME analytics platform (ver. 2.8.2), Zurich, Switzerland. https://www.knime.org/knime

[58] Xu D, Tian Y (2015). A comprehensive survey of clustering algorithms. Ann Data Sci.

[59] Estivill-Castro V (2002). Why so many clustering algorithms: a position paper. ACM SIGKDD Explor Newsl.

[60] Dunn JC (1973). A fuzzy relative of the ISODATA process and its use in detecting well-separated clusters”. J Cybern.

[61] Davies DL, Bouldin DW (1979). A cluster separation measure. IEEE Trans Pattern Anal Mach Intell.

[62] Hinkle DE, Wiersma W, Jurs SG (2002). Applied statistics for the behavioral sciences.

[63] Okada K (2013). Is Omega squared less biased? A comparison of three major effect size indices in one-way ANOVA. Behaviormetrika.

[64] Keselman HJ (1975). A Monte Carlo investigation of three estimates of treatment magnitude: epsilon squared. Eta squared and omega squared. Can Psychol Rev.

[65] Murtagh F (2014). Ward’s hierarchical agglomerative clustering method: Which algorithms implement Ward’s criterion?. J Classif.

[66] Dhillon IS, Parlett BN (2004). Orthogonal eigenvectors and relative gaps. SIAM J Matrix Anal Appl.

[67] Nguyen TD, Schmidt B, Kwoh CK (2014). SparseHC: a memory-efficient online hierarchical clustering algorithm. Proc Comput Sci.

[68] Python (2015) Python scientific software (ver. 2.7.3), Austin, TX. USA. https://www.python.org/

[69] R Core Team (2015) R: a language and environment for statistical computing. R Foundation for Statistical Computing, Vienna, Austria. https://www.R-project.org/

